# Inhibition of Inflammation and Regulation of AQPs/ENaCs/Na^+^-K^+^-ATPase Mediated Alveolar Fluid Transport by Total Flavonoids Extracted From *Nervilia fordii* in Lipopolysaccharide-induced Acute Lung Injury

**DOI:** 10.3389/fphar.2021.603863

**Published:** 2021-11-23

**Authors:** Shuomiao Yin, Meizhu Ding, Long Fan, Xuhua Yu, Ziyao Liang, Lei Wu, Zhiling Gao, Lin Lin, Yuanbin Chen

**Affiliations:** ^1^ Department of Intensive Care Unit, The First Affiliated Hospital of Anhui University of Chinese Medicine, Hefei, China; ^2^ Department of Respiratory Medicine, The Second Affiliated Hospital of Guangzhou University of Chinese Medicine, The Second Clinical College of Guangzhou University of Chinese Medicine and Guangdong Provincial Hospital of Chinese Medicine, Guangzhou, China; ^3^ Guangdong-Hong Kong-Macau Joint Lab on Chinese Medicine and Immune Disease Research, Guangzhou, China

**Keywords:** acute lung injury, nervilia fordii, flavonoids, lipopolysaccharide, aquaporins, epithelial sodium channel, Na^+^-K^+^-ATPase

## Abstract

**Aims:** The occurrence of vascular permeability pulmonary edema in acute lung injury (ALI) is related to the imbalance of alveolar fluid transport. Regulating the active transport of alveolar fluid by aquaporins (AQPs), epithelial sodium channels (ENaCs), and Na^+^-K^+^-ATPase can effectively reduce the edema fluid in the alveolar cavity and protect against ALI. We evaluated the therapeutic effects of total flavonoids, extracted from *Nervilia fordii* (TFENF), and investigated its potential mechanisms of alveolar fluid transport in a rat ALI model.

**Materials and methods:** A model of lipopolysaccharide (LPS, 5 mg/kg)-induced ALI was established in Sprague-Dawley (SD) rats through the arteriae dorsalis penis. SD rats were divided into six groups, including the vehicle, LPS model, TFENF (6 mg/kg, 12 mg/kg, 24 mg/kg), and dexamethasone group (DEX group, 5 mg/kg). The wet-to-dry (W/D) lung weight ratio, oxygenation index, and histopathological observation were used to evaluate the therapeutic effect of TFENF. The mRNA expression of *AQPs, ENaCs*, and pro-inflammatory cytokines was determined using real-time polymerase chain reaction, whereas protein expression was determined using immunohistochemistry. The *Na* ^*+*^ *-K* ^*+*^ *-ATPase* activity was assessed using enzyme-linked immunosorbent assay.

**Results:** LPS significantly stimulated the production of inflammatory mediators including tumor necrosis factor (TNF)-α and interleukin (IL)-1β, and disrupted the water transport balance in the alveolar cavity by inhibiting *AQPs/ENaCs/Na* ^*+*^ *-K* ^*+*^ *-ATPase*. Pretreatment with TFENF reduced the pathological damage and W/D ratio of the lungs and ameliorated the arterial blood oxygen partial pressure (PaO_2_) and oxygenation index. TFENF further decreased the mRNA level of *TNF-α* and *IL-1β*; increased the expression of *AQP-1, AQP-5, αENaC, and βENaC*; and increased *Na* ^*+*^ *-K* ^*+*^ *-ATPase* activity. Moreover, the regulation of *AQPs, βENaC,* and *Na* ^*+*^ *-K* ^*+*^ *-ATPase* and the inhibition of *TNF-α* and *IL-1β* by TFENF were found to be dose dependent.

**Conclusion:** TFENF protects against LPS-induced ALI, at least in part, through the suppression of inflammatory cytokines and regulation of the active transport capacity of *AQPs/ENaCs/Na* ^*+*^ *-K* ^*+*^ *-ATPase*. These findings suggest the therapeutic potential of TFENF as phytomedicine to treat inflammation and pulmonary edema in ALI.

## Introduction

Acute lung injury (ALI) is a life-threatening respiratory disease characterized by increased permeability of alveolar capillaries, effusion of protein fluid in the pulmonary alveoli, and hyaline membrane formation. A clinical epidemiology investigation revealed that the incidence of ALI in patients over 15 years of age was 86.2 cases per 100,000 person-years (Rubenfeld et al., 2005). ALI has a worrisome high mortality rate and is 40% in the United States (Johnson et al., 2010) and 50% in Europe (Brun-Buisson et al., 2004). There are no specific pharmacological strategies to treat ALI (Diamond et al., 2020; Matera et al., 2020), and current therapies are mainly based on the support of lung-protective mechanical ventilation (Fan et al., 2018; Chen et al., 2019). Therefore, the discovery of drugs for the management of ALI is challenging and should be actively pursued.

Inflammatory cytokine storm and the resultant impairment in gas exchange are the major causes of death in ALI. Pulmonary edema is the most important pathological characteristic in the progression of ALI (Matthay et al., 2005). Reabsorption of alveolar fluid ensures normal pulmonary gas exchange, which relies on the coordinated control of epithelial sodium channels (*ENaCs*), aquaporins (*AQPs*), and *Na* ^*+*^ *-K* ^*+*^ *-ATPase* (Ma et al., 2020). Na^+^ transport across epithelial cells is driven by *Na* ^*+*^ *-K* ^*+*^ *-ATPase*, which is present on the basolateral surface of epithelial cells. *Na* ^*+*^ *-K* ^*+*^ *-ATPase* provides 10 times the concentration of Na^+^ in the pulmonary interstitium than in epithelial cells. This Na^+^ gradient accelerates Na^+^ along with the transport of water from the alveolar spaces to the pulmonary interstitium and capillaries. *ENaCs* on the apical surface of alveolar epithelial cells and *AQPs* are the main channels for Na^+^ and water on epithelial and endothelial cells. It has been reported that *ENaCs* contribute to 40–60% of Na^+^ transport in rat lungs and up to 90% in mouse lungs (Mutlu et al., 2005). Briefly, *ENaCs*, *AQPs*, and *Na* ^*+*^ *-K* ^*+*^ *-ATPase* play pivotal roles in alleviating pulmonary alveolar edema (Wang et al., 2018). Regulation of the expression of *AQPs/ENaCs/Na + -K + -ATPase* has great significance in water transport in the lungs.

Inflammatory cytokine storm is a characteristic of ALI and one of the main factors interfering with the reabsorption of lung fluid during ALI, although *ENaC* is known to be directly regulated by the renin-angiotensin-aldosterone system (Zaika et al., 2013). Excessive release of pro-inflammatory cytokines, such as tumor necrosis factor (TNF)-α, destroying the sodium and chloride transport on epithelial barriers is reported to be the main reason for the inhibition of reabsorption of the lung fluid (Eisenhut et al., 2020). It has been reported that TNF-α induces alveolar epithelium apoptosis by p55-related death pathway and downregulates *Na* ^*+*^ *-K* ^*+*^ *-ATPase* in the rat colon via prostaglandin (PG) E2 (Markossian and Kreydiyyeh, 2005; Patel et al., 2013). Moreover, interleukin (IL)-6 is known to upregulate the expression of angiotensin and aldosterone or their receptors, which may indirectly increase *ENaC* expression (Wassmann et al., 2004). Therefore, anti-inflammation is an important therapeutic strategy in the management of ALI.

Traditional Chinese medicine, including Chinese herbal decoction and Chinese medicine injection, has potential advantages in the prevention and treatment of ALI and has been widely used in China. Evidence from systematic evaluation shows that Chinese medicine injection combined with conventional therapy can significantly reduce the inflammatory score and improve clinical efficacy (Chen et al., 2019). *N. fordii* (Hance) Schltr. a Chinese herbal medicine, is widely used to treat infectious diseases in China. In traditional Chinese medicine, it is used for clearing heat toxins, inducing diuresis for edema removal, and moistening the lung to prevent coughing. It also exerts antiviral, antibacterial, and anti-inflammatory effects (Chen et al., 2013). Flavonoids are found in many fruits, vegetables, and herbs, and are known to possess antiaging, anti-inflammatory, and anti-cancer properties (Zhou et al., 2019). Flavonoids are the main components of *N. fordii*. To date, more than 20 flavonoids have been identified in *N. fordii*. (Qiu et al., 2013).

We previously showed that pretreatment with *N. fordii* water decoction could regulate aquaporin and reduce pulmonary edema caused by ALI (Xu et al., 2010). The protective effect of a *N. fordii* injection, containing total flavonoids and amino acids, against ALI through the inhibition of inflammation has been confirmed (Xu et al., 2014). However, the efficacy of the total flavonoids from *N. fordii* (TFENF), which are the main components of *N. fordii* injection, on ALI and the molecular mechanism underlying their effects are currently unknown. Thus, we assessed the therapeutic effects of TFENF using an LPS-induced ALI model. We evaluated the effects of TFENF on the release of inflammatory cytokines and expression of *AQPs/ENaCs/Na* ^*+*^ *-K* ^*+*^ *-ATPase*, and investigated its potential mechanisms in alveolar fluid transport.

## Materials and Methods

### Plant Materials and Preparation of TFENF

Wild *N. fordii* was collected from Guangxi Province, China, and was confirmed as the whole herbal medicine, *N. fordii* (Hance) Schltr, by the Chinese pharmacist Yunfeng Huang from the Guangxi Institute of Traditional Chinese Medicine and Pharmaceutical Science. *N. fordii* was cut into pieces, placed in a leakage cylinder, and soaked in 95% ethanol. The ethanol extract was collected and concentrated to remove the alcohol. The resultant *N. fordii* extract was poured into glass columns containing AB-8 porous resin, washed with pure water until the outflowing liquid was colorless, and was then washed with 70% ethanol until the eluent was colorless. The ethanol eluate was collected, concentrated to remove alcohol, and then diluted with pure water. The residual liquid was added into the polyamide column, washed until colorless, and eluted with 70% ethanol until colorless. The eluent was collected and concentrated into TFENF powder.

**FIGURE 1:**
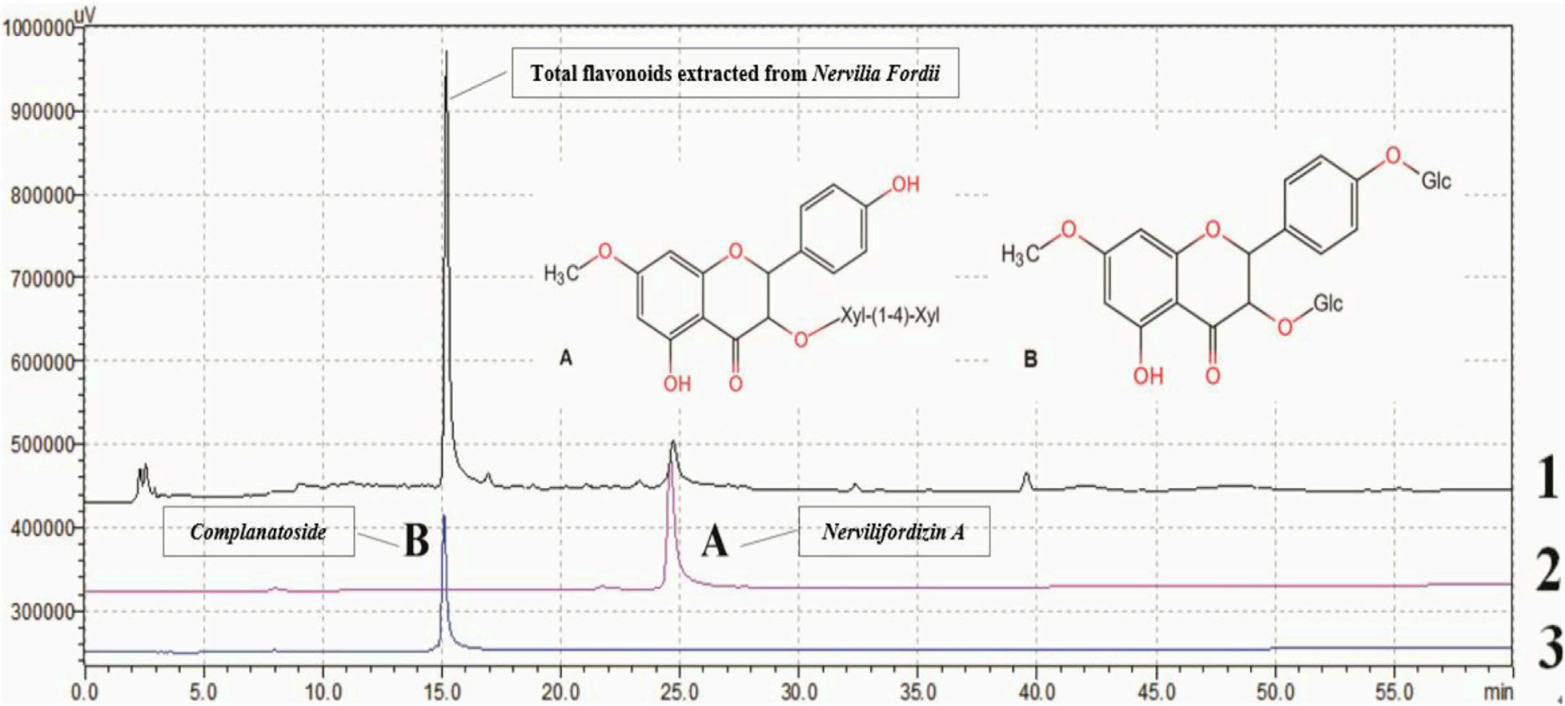
Chemical profile of TFENF analyzed using HPLC. 1. Total flavonoids extracted from *Nervilia fordii*; 2 **(A)** *Nervilifordizin A*; 3 **(B)** *Complanatoside*.

TFENF composition was determined by high-performance liquid chromatography (HPLC) using an Agilent-1260 HPLC System equipped with a PhenomenexLuna 5 μ PFP column (4.6 × 250 mm, 5 μm). A mobile phase of acetonitrile and 0.2% methane acid was used for the analysis and the elution profile for TFENF is shown in Table 1. Two mixed standard reference substances were selected and identified by Dr. Zhu Chenchen from the Institute of Clinical Pharmacology of Guangzhou University of Chinese Medicine to have a purity >98.5%. The standard reference compounds are shown in Table 2 and their chemical structural formula is depicted in Figure 1.

**TABLE 1:** HPLC elution profile for TFENF(flow rate: 1 ml/min).

Time (min)	% of acetonitrile	% of 0.2% methane acid
0	15	85
21	19	81
40	23	77
50	50	50
60	100	0

**TABLE 2:** The standard reference substance for TFENF.

Name	Chemical Formula	Formula Weight
Nervilifordizin A	C_28_H_32_O_16_	624
Complanatoside	C_28_H_32_O_16_	624

### Animals and Experimental Design

Male, 5–7 week-old, SD rats weighing 150–200 g were purchased from the Animal Laboratory Center of the Guangzhou University of Chinese Medicine. All the animals were allowed to adapt to the environment for 5 days prior to experiments. They were fed food and water under sterile pathogen-free conditions and maintained at 23–26°C at a relative humidity of 40–60% and subjected to a standard 12 h/12 h light/dark cycle. Anesthetic drugs and other necessary measures were used to reduce animal suffering during all experimental procedures. All standardized processes were carried out in accordance with the Regulations of Experimental Animal Administration issued by the Ministry of Science and Technology of the People’s Republic of China.

Rats were divided into six groups, with six rats per group as follows: Vehicle, LPS model, Low dose (6 mg/kg) of TFENF (TFENF-L group), Middle dose (12 mg/kg) of TFENF (TFENF-M group), High dose (24 mg/kg) of TFENF (TFENF-H group), and dexamethasone (DEX group, 5 mg/kg). TFENF and dexamethasone dosages were based on our previous research (Yinji et al., 2014) and preliminary experiments. Rats in the drug-intervention groups were pretreated with TFENF or DEX through intraperitoneal injection for 7 days, whereas those in the vehicle and LPS model groups were injected with an equal amount of vehicle. One hour after the last drug pretreatment, rats in the experimental groups were injected with 5 mg/kg LPS or 0.1 ml normal saline through the vena dorsalis penis, while those in the Vehicle group were injected with an equal volume of vehicle. Rats were sacrificed 6 h after the LPS injection.

### Histopathological Evaluation

The right middle lung tissue was removed and fixed with 10% formaldehyde solution for 3 days. The lung tissue-biopsy material was dehydrated using a serial alcohol gradient, embedded in paraffin, and prepared into 4 μm-thick sections. After dewaxing with xylene and hydration with ethanol, lung tissue sections were washed with phosphate-buffered saline (PBS) and the sections were stained with hematoxylin-eosin (H&E). Next, the sections were sealed with neutral balsam and histopathological changes were observed using a light microscope (Olympus, Japan). The lung injury scores of each rat were calculated in five random fields (×400) for histopathological evaluation and graded as follows: 0 = no injury, 1 = slight injury (25%), 2 = moderate injury (50%), 3 = severe injury (75%), and 4 = very severe injury (almost 100%) (Deng et al., 2012).

### Blood Gas Analysis

Blood gas analysis including the oxygenation index (OI) and partial pressure of oxygen in the artery (PaO_2_) was obtained using a blood gas analyzer (Siemens RAP500, Germany). Samples for blood gas analysis were drawn from the aorta abdominals and immediately sent for evaluation. The OI was calculated using the following equation: OI = PaO_2_/21%.

### Wet-To-Dry (W/D) Lung Weight Ratio

The W/D ratio was used to evaluate lung edema in ALI. The left lung tissue was weighed to obtain the wet lung weight, placed in an oven at 65°C for 72 h, and reweighed to obtain the dry lung weight. The W/D ratio was calculated using the following equation: W/D = Wet lung weight/Dry lung weight.

### Reverse-Transcription Polymerase Chain Reaction (RT-PCR)

Total RNA was isolated from the lung tissues using NucleoZOL reagent (MN, Duren, Germany) and a reverse transcription kit (Lot#AI20778A, TaKaRa, Japan) was used to transcribe the RNA to cDNA. Reverse-transcription PCR expression analysis was performed using an ABI 7500 PT-PCR System (Applied Biosystems, United States) and SYBR Premix Ex Taq (Lot#RR820A, TaKaRa, Japan). Primers used for RT-PCR are shown in Table 3.

**TABLE 3:** PCR primers for different observation indices.

Gene target	Forward primer (5–3′)	Reverse primer (5–3′)
*αENaC*	F-TACATGGGGTGGTGGAACTTG-R	R-GAAGGACTGGAAGATCGGCT-F
*βENaC*	F-GGCCACTAGCTGATGACAGT-R	R-CCGTACCATCGAGGAATCGC-F
*γENaC*	F-GGAGCCAAGGTGCTTATCCA-R	R-GGGAGTAGGCAGCGTTGTAG-F
*AQP-1*	F-CGGTCAGTGGTAGCCAGAAC-R	R-ATCCTCCGGGCTGTCATGTA-F
*AQP-5*	F-GGTTTATTGGGAAGCGCCAG-R	R-AGGGATAGATGGCTCACGGA-F
*TNF-α*	F-GCTTGGTGGTTTGCTACGAC-R	R-GCTTGGTGGTTTGCTACGAC-F
*IL-1β*	F-GTGCCGTCTTTCATCACACA-R	R-ACAAAAATGCCTCGTGCTGTC-F
*β-actin*	F-AGGGTGTAAAACGCAGCTCA-R	R-GATCAAGATCATTGCTCCTCCTG-F

### Immunohistochemistry

The right middle lung sections from each group were deparaffinized with xylene, rehydrated using gradient ethanol, and incubated in 3% H_2_O_2_ for 15 min at 37°C. The sections were rinsed 3 times (5 min per rinse) in PBS, blocked with bovine serum albumin for 30 min, and incubated with primary antibodies at 4°C for 24 h. After washing 3 times (5 min per rinse) with PBS, rabbit IgG was added to the sections and incubated for 30 min, followed by staining with DBA, counterstaining with hematoxylin, dehydration with gradient ethanol, vitrification with xylene, and lastly, sealing with neutral balsam.

### Na^+^-K^+^-ATPase Activity Assay

Lung tissue homogenate was prepared using normal saline according to the weight-to-volume ratio. After centrifugation, the supernatant was removed, and the lung protein concentration was determined using an enzyme-labeled meter. ATP enzyme test kits (Jiancheng Company, Nanjing, China) were used to assay *Na* ^*+*^ *-K* ^*+*^ *-ATPase* activity following the manufacturer’s instructions.

### Statistical Analysis

Data are presented as the mean ± standard error of the mean (S.E.M.). One-way analysis of variance (ANOVA) was used to analyze differences between groups. A 5% level of probability was considered significant for all analyses. SPSS 20.0 was used to analyze data and the graphs were generated using GraphPad Prism 7 (GraphPad Software Inc., La Jolla, CA, United States). *p* < 0.05 was considered statistically significant.

## Results

### Qualitative Analysis of TFENF Using HPLC

TFENF was analyzed using HPLC with two different reference standard compounds. As shown in Figure 1, over three peaks were generated within 40 min. At 15 min, the first chromatographic peak appeared in the sample and was found to correspond to that of *complanatoside*. The second peak appearing in the sample at 25 min was found to correspond with that of *nervilifordizin A*. In brief, a comparison of individual peak retention times with those of the reference standard compounds resulted in the identification of the two compounds, *nervilifordizin A* and *complanatoside*.

### TFENF Reduced the Severity of Pathological Injury of Lung Tissue

**FIGURE 2:**
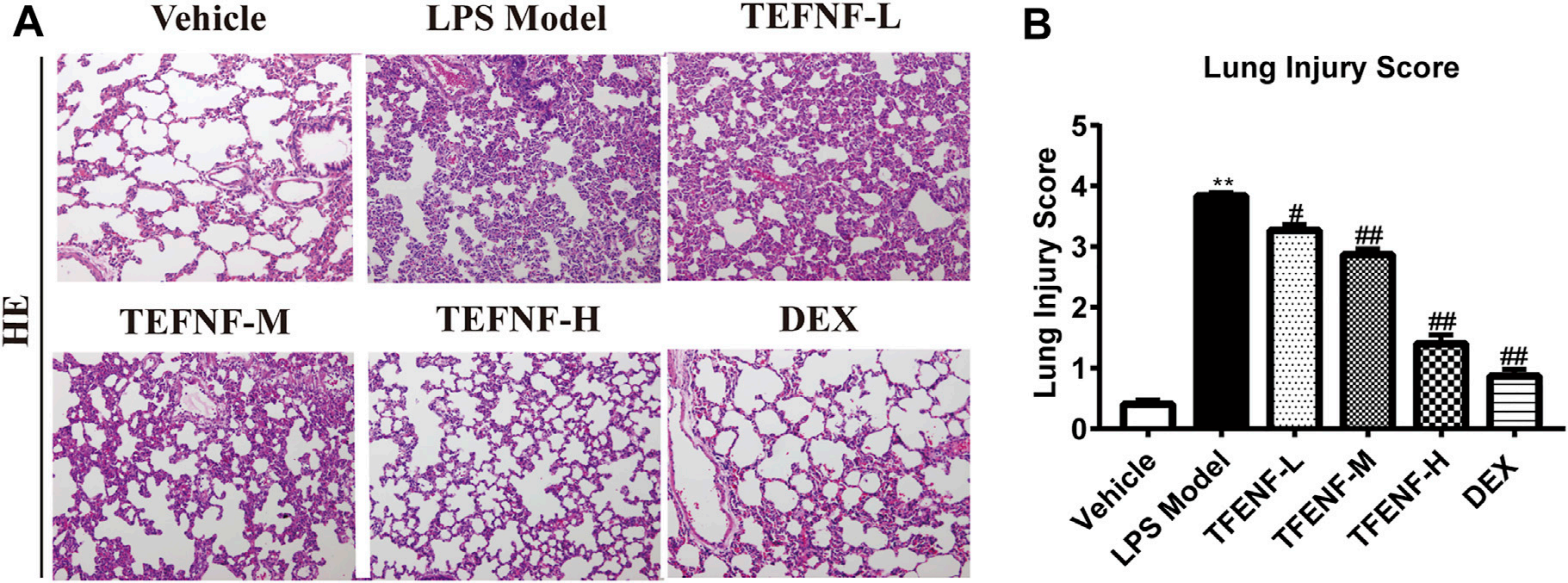
TFENF protected against pulmonary pathological damage and inflammatory cell infiltration in LPS-induced ALI. Effect of TFENF on LPS-induced changes in lung histology **(A)** and lung injury score **(B)** 6 h after LPS challenge or saline treatment (*n* = 6 per group). Lung sections stained using H&E (×200). Lung injury scores are presented as mean ± S.E.M. ^**^ *p* < 0.01 compared with the vehicle group; ^#^ *p* < 0.05 compared with the LPS model group; ^*##*^ *p <* 0.01 compared with the LPS model group.

As shown in Figure 2, no histopathological changes were observed in the vehicle group **(** Figure 2A **)**, the alveolar morphology was uniform, and there was no alveolar collapse or neutrophil infiltration. The bronchial wall was smooth and no hyperemia and edema were observed in the interstitial edema. However, in the LPS model group, a series of pathological changes were observed including the pulmonary interstitial thickening and alveolar collapse, and large numbers of neutrophils infiltrating the pulmonary interstitium **(** Figure 2A **)**. After pretreatment with TFENF and dexamethasone, pulmonary interstitial thickening, alveolar collapse, and neutrophil invasion were significantly reduced, the lung tissue morphology was closer to that of the Vehicle group, and the edema of lung interstitium significantly decreased (Figure 2A). The lung injury score showed that the model group scored higher than the Vehicle group after pretreatment with TFENF and dexamethasone, and the lung injury score decreased significantly compared with that of the model group (Figure 2B).

### TFENF Improved PaO_2_ and OI in LPS-Induced ALI

**FIGURE 3:**
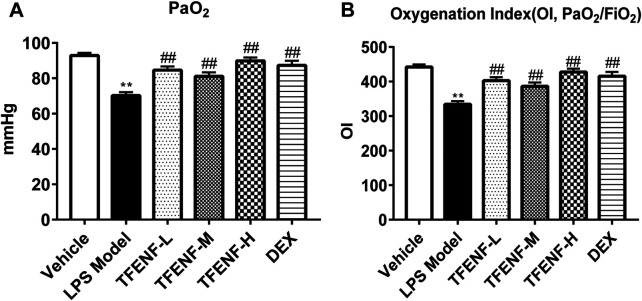
TFENF ameliorated PaO_2_ and IO of arterial blood gas analysis in LPS-induced ALI. Rat arterial blood gas analysis to determine PaO_2_ **(A)** and IO **(B)** 6 h after LPS-induced ALI or saline treatment (*n* = 6 per group). Data are presented as mean ± S.E.M. ^**^ *p* < 0.01compared with the vehicle group; ^*#*^ *p <* 0.05 compared with the LPS model group; ^*##*^ *p <* 0.01 compared with the LPS model group.

As shown in Figure 2A, stimulation with LPS destroyed the alveolar integrity, caused pulmonary edema, increased pulmonary capillary permeability, and, consequently, led to a reduction in pulmonary ventilation and ventilation function. The decrease in OI is one of the main parameters when diagnosing ALI. Arterial blood gas analysis was performed to confirm the role of TFENF oxygenation improvement in LPS-induced ALI. PaO_2_ and OI were decreased in the LPS model group; however, the degree of PaO_2_ and O/I were ameliorated in the TFENF and DEX groups compared with those in the LPS model group (*p <* 0.01) **(** Figure 3 **)**.

### TFENF Reduced Lung W/D Ratio in LPS-Induced ALI

**FIGURE 4:**
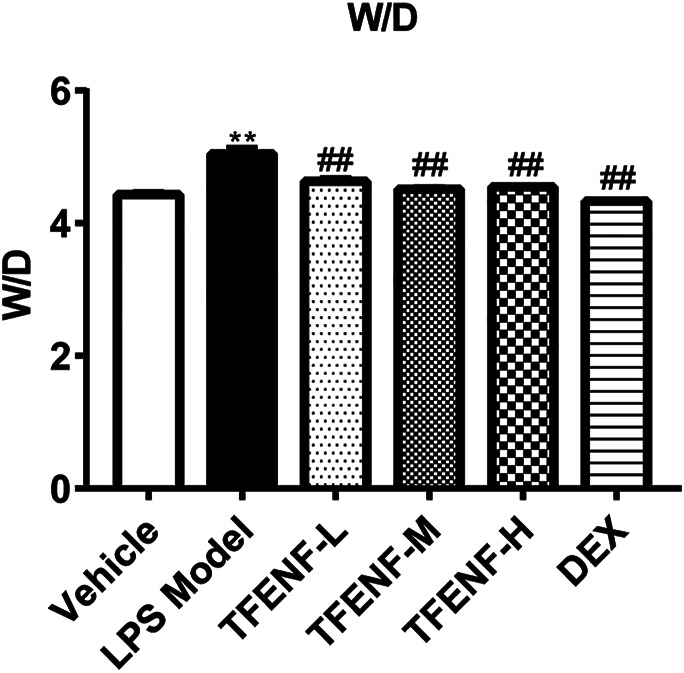
Effects of TFENF on the W/D ratio in LPS-induced ALI. Effects of TEFNF on the W/D ratio in rat lungs 6 h after LPS challenge (*n* = 6 per group). Data are presented as mean ± S.E.M. ^**^ *p <* 0.01 compared with the vehicle group; ^*##*^ *p <* 0.01 compared with the LPS model group.

The lung W/D ratio was calculated to evaluate the potential protective effect of TFENF on pulmonary edema. As shown in Figure 4, compared with that in the vehicle group, stimulation with LPS caused a significant increase in the lung W/D ratio in the LPS model group (*p <* 0.01). However, compared with that in the LPS model group, the lung W/D ratio declined significantly after TFENF intervention (*p <* 0.01), similar to that in the DEX group. Moreover, with an increased dosage of TFENF in the intervention group, the W/D ratio was determined to be closer to that of the vehicle group.

### TFENF Decreased the mRNA Expression Level of TNF-α and IL-1β

**FIGURE 5:**
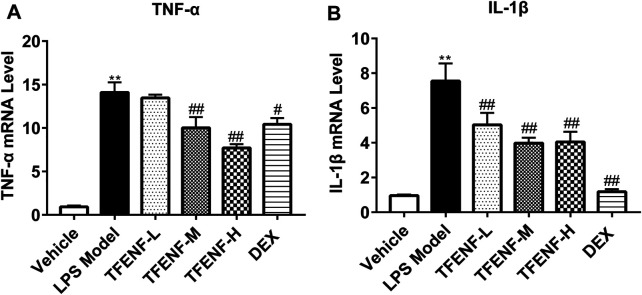
TFENF suppressed the expression of pro-inflammatory cytokines in LPS-induced ALI. Measurement of mRNA levels of TNF-α **(A)** and IL-1β **(B)** in lung tissue 6 h after LPS-induced ALI or saline treatment (*n* = 6 per group). Data are presented as mean ± S.E.M. ^**^ *p <* 0.01 compared with the vehicle group; ^*#*^ *p < 0.05* compared with the LPS model group; ^*##*^ *p <* 0.01 compared with the LPS model group.

To determine the anti-inflammatory effect of TFENF pretreatment on LPS-induced ALI, we evaluated the gene expression of *TNF-α* and *IL-1β* in lung tissue samples from different groups. *TNF-α* and *IL-1β* mRNA expression increased significantly in the LPS model group (Figure 5). Compared with the LPS model group, the gene expression of *TNF-α* and *IL-1β* decreased after TFENF pretreatment, especially in the TFENF-M and TFENF-H groups (*p <* 0.01). Similarly, a significant decline was observed in the DEX group.

### TFENF Increased the mRNA Expression Level of *AQPs* and *ENaCs*

**FIGURE 6:**
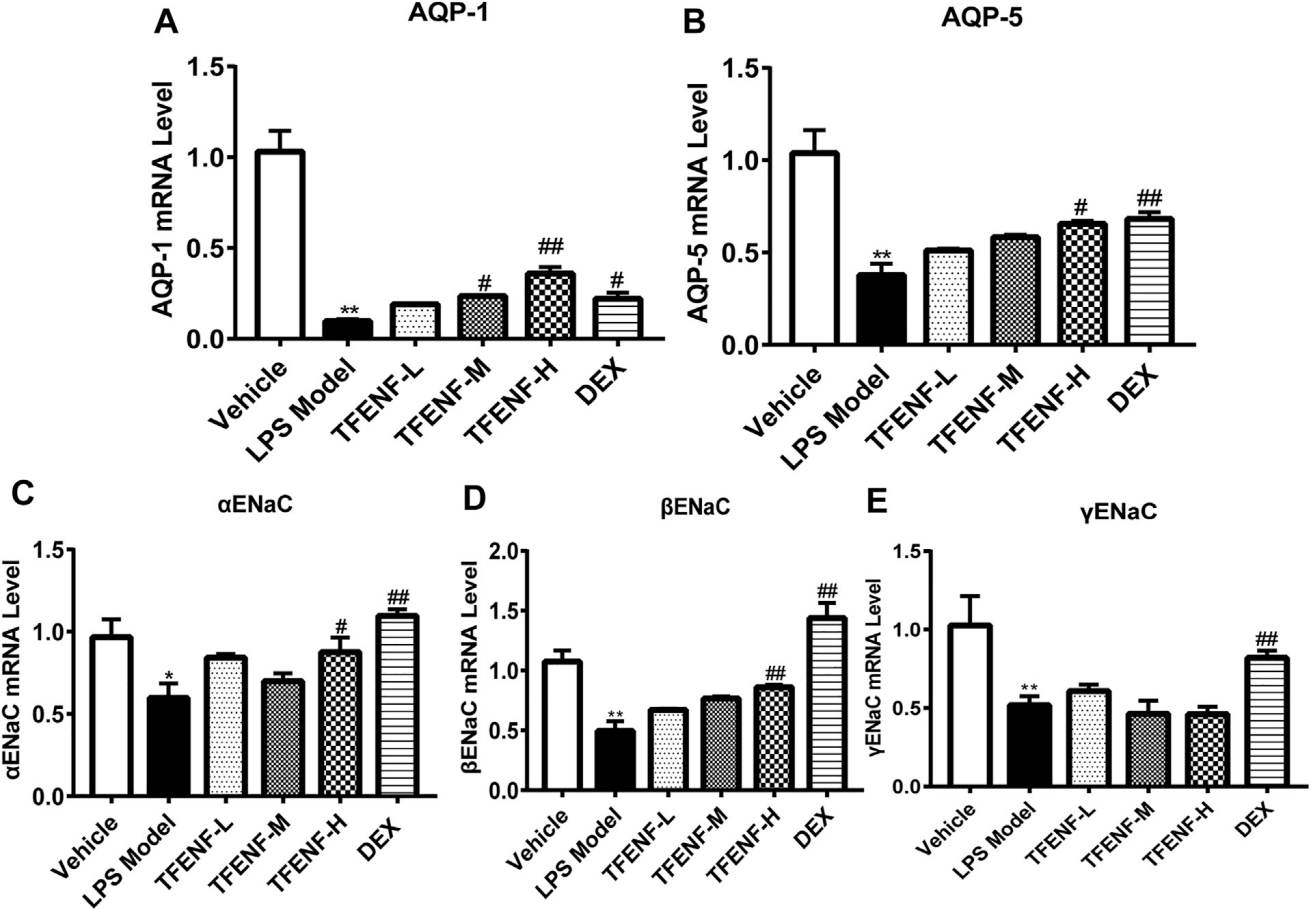
TFENF increased the mRNA expression of AQPs and ENaC in LPS-induced ALI. mRNA expression of AQP-1 **(A)**, AQP-5 **(B)**, αENaC **(C)**, βENaC **(D)**, and γENaC **(E)** in lung tissue 6 h after LPS-induced ALI or saline treatment (*n* = 6 per group). Data are presented as mean ± S.E.M. ^*^ *p <* 0.05 compared with the vehicle group; ^**^ *p <* 0.01 compared with the vehicle group; ^*#*^ *p < 0.05* compared with the LPS model group; ^*##*^ *p <* 0.01 compared with the LPS model group.

AQPs and ENaCs play key roles in LPS-induced ALI lung edema pathogenesis. Compared with that in the vehicle group, LPS stimulation suppressed the mRNA expression of *AQP-1, AQP-5, αENaC*, *βENaC*, and *γENaC* in the LPS model group (Figure 6). The mRNA expression of *AQP-1, AQP-5,* and *βENaC* increased in the TFENF pretreatment groups **(** Figures 6A,B,D **)**. With an increase in TFENF dosage, the gene expression in the three groups (TFENF-L, TFENF-M, and TFENF-H) exhibited an increasing trend. This trend was not observed in terms of *αENaC* gene expression **(** Figure 6C **)**. In the *AQP-1* group, the TFENF-M and TFENF-H groups exhibited differences compared with the LPS model group, but in the *AQP-5 and ENaC* groups, the differences were only observed in the TFENF-H group when compared with the LPS model group. However, TFENF pretreatment had no obvious effect on *γENaC* (Figure 6E). In the DEX group, the mRNA levels of *AQP-1, AQP-5, αENaC, βENaC*, and *γENaC* increased significantly compared with those of the LPS model group.

### TFENF Promoted the Protein Expression of *AQPs* and *ENaCs*

**FIGURE 7:**
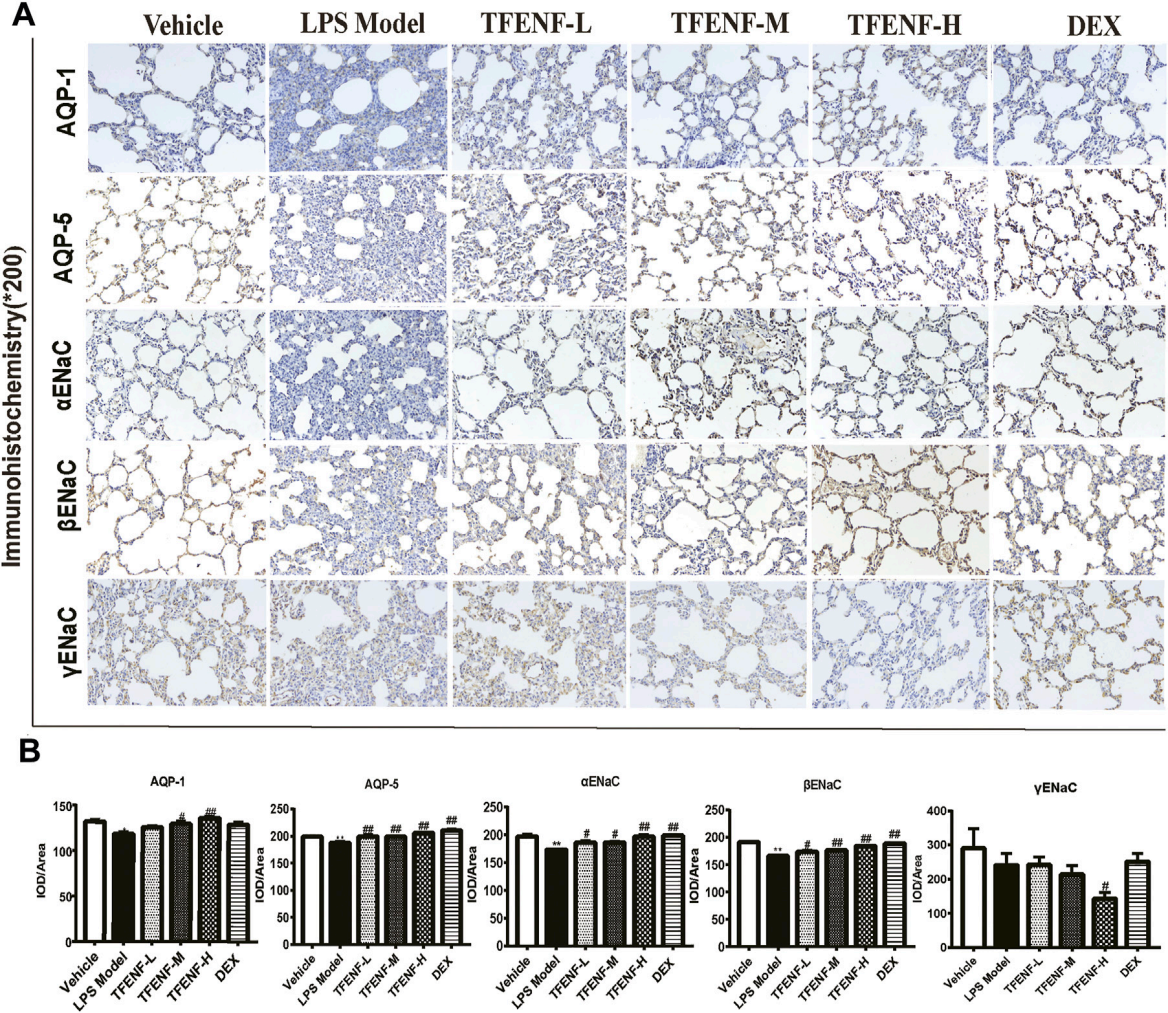
TFENF promoted the protein expression of *AQPs* and ENaCs in LPS-induced ALI. Effect of TFENF on the protein expression of *AQP-1, AQP-5*, *αENaC, βENaC*, and γENaC in rat lung tissue 6 h after LPS-induced ALI or saline treatment (*n* = 6 per group) (immunohistochemistry staining, magnification ×200). Representative photographs of immunohistochemistry assay **(A)** and quantitative analysis of immunohistochemistry results **(B)**. Data are presented as mean ± S.E.M. The numbers of cells expressing *AQP-1, AQP-5, αENaC*, and *βENaC* are significantly decreased in LPS-induced ALI compared with those in the vehicle group. Meanwhile, the numbers of cells expressing *AQP-1*, *AQP-5*, *αENaC*, and *βENaC* are significantly increased after TFENF treatment. Brown immunostained cells are positive. ^*^ *p <* 0.05 compared with the vehicle group; ^**^ *p <* 0.01 compared with the vehicle group; ^*#*^ *p <* 0.05 compared with the LPS model group; ^*##*^ *p <* 0.01 compared with the LPS model group.

Immunohistochemical analysis was used to determine the effect of pretreatment with TFENF on the subcellular distribution of *AQP-1, AQP-5, αENaC, βENaC,* and *γENaC*. Positively immunostained cells appeared brown. As shown in Figure 7, compared with that in the LPS model group, *AQP-1, AQP-5, αENaC,* and *βENaC* protein expression increased after treatment with different doses of TFENF, and this difference was especially prominent in the TFENF-H group (*p <* 0.01). However, the expression of *γENaC* in the TFENF-H group was significantly lower than that in the LPS model group (*p <* 0.05). The protein expression levels of *AQP-5, αENaC*, and *βENaC* in the DEX group were different compared with those in the LPS model group (*p <* 0.01), except for *AQP-1* protein expression.

### TFENF Increased the Enzymatic Activity of Na^+^-K^+^-ATPase

**FIGURE 8:**
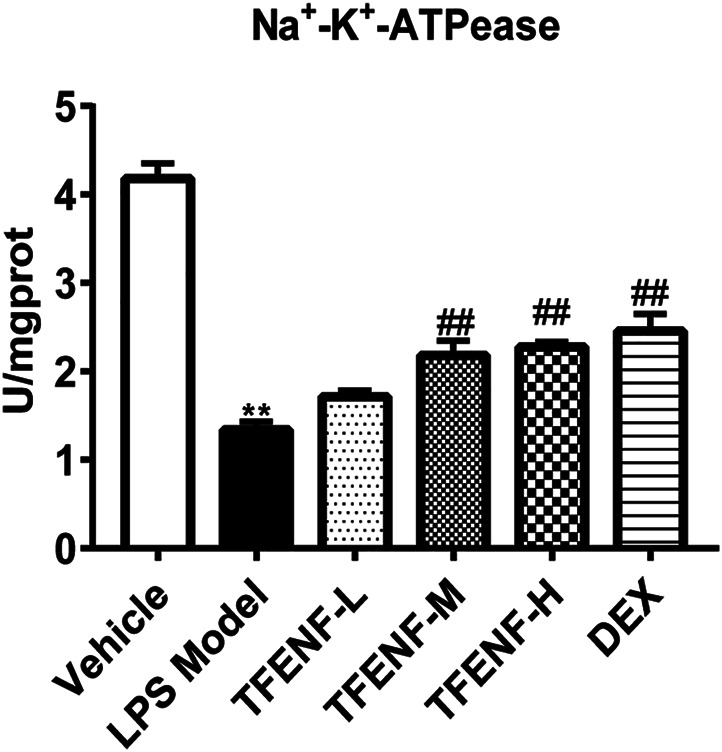
TFENF increased the enzymatic activity of *Na* ^*+*^ *-K* ^*+*^ *-ATPase* in LPS-induced ALI. Increase in the enzymatic activity of *Na* ^*+*^ *-K* ^*+*^ *-ATPase* by TFENF in rat lung tissue 6 h after LPS-induced ALI or saline treatment (*n* = 6 per group). Data are presented as mean ± S.E.M. ^**^ *p <* 0.01 compared with the vehicle group; ^*##*^ *p <* 0.01 compared with the LPS model group.

To determine whether TFENF affected the enzymatic activity of *Na* ^*+*^ *-K* ^*+*^ *-ATPase*, we measured *Na* ^*+*^ *-K* ^*+*^ *-ATPase* activity in lung tissue. The results showed that LPS inhibited *Na* ^*+*^ *-K* ^*+*^ *-ATPase* activity whereas *Na* ^*+*^ *-K* ^*+*^ *-ATPase* activity in the TFENF-administered groups increased, and a significant difference was observed between the TFENF-L and LPS model groups. However, compared with the LPS model group, TFENF-M, TFENF-H, and DEX groups showed a significant increase in *Na* ^*+*^ *-K* ^*+*^ *-ATPase* activity (*p <* 0.01) (Figure 8).

## Discussion

Various factors, within or outside the lung, likely induce ALI. Damage to the pulmonary capillary endothelial and alveolar epithelial cells can lead to increased permeability of the capillary wall, imbalance in alveolar fluid transport, influx of a large amount of protein-rich fluid into the alveoli, and acute pulmonary edema, which cannot be explained by cardiogenic pulmonary edema (Jiang et al., 2015). In ALI, pulmonary ventilation and air-exchange functions are severely impaired, and the ventilation/blood flow ratio is imbalanced as alveoli are filled with a large amount of fluid. The blood oxygen saturation cannot be improved despite the increased inhaled oxygen. Therefore, the OI decreases in ALI, and the decrease is positively related to disease severity. In addition, large numbers of inflammatory factors accumulate in the lung and trigger an inflammatory waterfall effect in ALI, leading to acute pulmonary edema, the main pathological feature of ALI.

ALI has an acute onset, complex mechanism, rapid progression, and high mortality, which is challenging in critical illness in a clinical setting. As a traditional Chinese herbal medicine, *N. fordii* has been widely used to treat pneumonia, sepsis, and other infectious diseases. Recently, some studies have confirmed the antibacterial (Xiong et al., 2014), antioxidant (Zhou et al., 2018), anti-inflammatory (Qiu et al., 2013; Jiao et al., 2014), antipyretic, and analgesic effects of *N. fordii* (Xie, 2008; Mei et al., 2011). Our previous study has reported the protective effect of *N. fordii* against ALI. The *N. fordii* water decoction promotes the expression of AQP-1 and AQP-5 in ALI and increases the clearance and transfer of lung water, improves water metabolism, and reduces pulmonary edema (Xu et al., 2010). Additionally, the total flavonoids and amino acids have been extracted as an injectable, which have been shown to exhibit protective effects against lung injury by inhibiting the overexpression of TNF-α and IL-6 (Xu et al., 2014). Furthermore, in *vivo* studies, *N. fordii* injection has demonstrated significant inhibition of the proliferation of J774 macrophages and production of TNF-α and IL-6 in the cell supernatant (Xu et al., 2014). However, there are only a few reports on the effect of *N. fordii* or its main components on the regulation of alveolar fluid transport.

In this study, a rat model of LPS-induced ALI was established to elucidate the possible mechanism of TFENF in the inflammatory response and alveolar fluid transport. We showed that LPS significantly stimulated the production of inflammatory mediators and disrupted the balance of water transport in the alveolar cavity. To our knowledge, this is the first study to report changes in different key links (*AQPs/ENaCs/Na* ^*+*^ *-K* ^*+*^ *-ATPase*) in the imbalance of the active transport of alveolar fluid in an ALI model. Moreover, pretreatment with TFENF reduced the pathological damage and W/D ratio and improved PaO_2_ and OI. TFENF also decreased the mRNA levels of *TNF-α* and *IL-1β* in a dose-dependent manner, increased both the mRNA and protein levels of *AQP-1, AQP-5, αENaC,* and *βENaC* (but not *γENaC*), and upregulated *Na* ^*+*^ *-K* ^*+*^ *-ATPase* activity. All of the above results demonstrated that TFENF reduced the transcription of pro-inflammatory cytokines in the lungs and alleviated lung edema induced hypoxima by increasing the expression of aquaporins and *ENaCs*, and Na^+^-K^+^-ATPase activity. Meanwhile, both mRNA and protein levels of *AQP-1, AQP-5, αENaC,* and *βENaC* were upregulated by TFENF, which indicated that TFENF upregulated the expression of *AQPs* and *ENaCs* at the transcriptional level. Moreover, our results indicated that TFENF selectively increased *αENa* and *βENaC* expression but not that of *γENaC*, demonstrating the possibility that α and β but not the γ subunit are the regulation targets of TFENF.

However, why TFENF is unable to increase *γENaC* expression is an interesting topic. Studies have demonstrated that the three subunits are usually synthesized in a differential fashion in cells that express endogenous *ENaC*, with one or two subunits expressed constitutively (Weisz et al., 2003; Peters et al., 2014). This finding suggests that *ENaC* subunits are regulated in a non-coordinated manner (Kim et al., 2018). On the other hand, whether TFENF is selective in the regulation of *ENaC* subunits needs further discussion.

LPS, the main component of the outer membrane of Gram-negative bacteria (Maldonado et al., 2016), stimulates neutrophils, macrophages, and other immune cells to produce different mediators including pro-inflammatory cytokines such as TNF-α and IL-1β, and the anti-inflammatory cytokines IL-10 and TGF-β (Fligiel et al., 2006). Excessive release of these cytokines recruits polymorphonuclear neutrophils to the injury site and leads to ALI. Therefore, it is crucial to suppress the excessive production of pro-inflammatory factors to control ALI progression (Idell, 2001; Toews, 2001).

Intravenous LPS-induced ALI is a commonly used modeling method. The pathological features of ALI are acute pulmonary edema, accompanied by a decrease in OI, and pathological damage to the lung tissue. W/D is a common indicator to evaluate the degree of pulmonary edema in an ALI model. In our study, an increase in the W/D ratio and a decrease in OI were observed in the LPS model group, in addition to significant pathological damage. Furthermore, a large increase in pro-inflammatory cytokines including TNF-α and IL-1β were observed, which indicated ALI. Our results are consistent with those of previous studies that demonstrated that pro-inflammatory cytokines play an important role in LPS-induced inflammatory responses (Yang et al., 2018; Ding et al., 2019). TFENF intervention could reduce pathological damage and W/D ratio, and improve PaO_2_ and OI in ALI. The main mechanism is the inhibition of the production of pro-inflammatory cytokines (TNF-α and IL-1β).

The mechanism of active transport of alveolar fluid in pulmonary edema is complex and involves multiple key links or pathways. Currently, it is clear that the regulation of *AQPs, ENaC*, and *Na* ^*+*^ *-K* ^*+*^ *-ATPase* is a typical representation and plays a vital role in active transport. Previous studies have shown that the expression of *AQPs* and *ENaCs* was decreased and the activity of *Na* ^*+*^ *-K* ^*+*^ *-ATPase* was suppressed in LPS-induced models of ALI (Hasan et al., 2014; He et al., 2015; Jiang et al., 2015). Our study also confirmed that an imbalance in alveolar fluid transport was related to the inhibition of *AQP-1, AQP-5, αENaC, βENaC, γENaC*, and *Na* ^*+*^ *-K* ^*+*^ *-ATPase*.

Some studies have reported the association between pro-inflammatory cytokines and active transport of alveolar fluid. TNF-α induces a decrease in *AQP-1* and *AQP-5* (Mezzasoma et al., 2013), whereas IL-1β inhibition can reverse the decrease of *AQP-1* and *AQP-5* (Yu et al., 2018). Previous evidence has also confirmed that TNF-α and IL-1β directly downregulate *ENaC* levels and expression in alveolar epithelial cells and their profound influence on the capacity of alveolar epithelial cells to transport sodium (Dagenais et al., 2004; Matalon et al., 2015; Wynne et al., 2017). Moreover, TNF-α is known to downregulate *Na* ^*+*^ *-K* ^*+*^ *-ATPase* and *Na* ^*+*^ *-K* ^*+*^ *2Cl* ^*-*^ cotransporter in the renal cortex and medulla and in the colon of rats via PGE2 (Markossian and Kreydiyyeh, 2005; keydiyyeh and Markossian, 2006), whereas IL-1β has been shown to inhibit *Na* ^*+*^ *-K* ^*+*^ *-ATPase* activity and protein expression in cardiac myocytes (Kreydiyyeh et al., 2004). However, there are only a few studies that focus on pulmonary edema. In brief, stimulation of inflammatory cytokines may inhibit the expression and activity of *AQPs*, *ENaCs*, and *Na* ^*+*^ *-K* ^*+*^ *-ATPase* in alveolar epithelial cells. In the current study, different doses of TFENF were found to promote alveolar fluid transport by regulating *AQP-1, AQP-5, αENaC, βENaC*, and *Na* ^*+*^ *-K* ^*+*^ *-ATPase* to reduce pulmonary edema. The underlying mechanism may be associated with the inhibition of the pro-inflammatory cytokines, TNF-α and IL-1β.

We determined the composition of TFENF using HPLC. Two standard reference compounds *nervilifordizin A* and *complanatoside* were used. The composition of the *N. fordii* is complex and the identification of total flavonoids is a useful approach to provide an objective and accurate basis for future animal- or cell-based experiments.

We found that *N. fordii* not only inhibited the expression of inflammatory factors but also promoted the expression of *AQPs*, *ENaCs*, and *Na* ^*+*^ *-K* ^*+*^ *-ATPase* in LPS-induced ALI in rats. Thus, *N. fordii* demonstrates the potential for multi-target effects in ALI treatment. Our findings may provide new avenues for the discovery of effective drugs to treat ALI.

## Conclusion

Using *in vivo* experiments, we demonstrated that pretreatment with TFENF can protect against pulmonary edema caused by LPS-induced ALI in rats. The protective mechanism is associated with the regulation of *AQPs/ENaCs/Na* ^*+*^ *-K* ^*+*^ *-ATPase*, which may be associated with the inhibition of pro-inflammatory cytokines. These findings demonstrate that TFENF could be a potential therapeutic phytomedicine to treat inflammation and pulmonary edema in ALI.

## Data Availability

The raw data supporting the conclusions of this article will be made available by the authors, without undue reservation.
